# The complete mitochondrial genome of the hermaphroditic freshwater mussel *Anodonta cygnea* (Bivalvia: Unionidae): *in silico* analyses of sex-specific ORFs across order Unionoida

**DOI:** 10.1186/s12864-018-4583-3

**Published:** 2018-03-27

**Authors:** E. E. Chase, B. M. Robicheau, S. Veinot, S. Breton, D. T. Stewart

**Affiliations:** 1 Department of Biology, Acadia University, Wolfville, NS Canada; 2 Department of Biology, Dalhousie University, Halifax, NS Canada; 3 Département de Sciences Biologiques, Université de Montréal, Montréal, QC, Canada

**Keywords:** Doubly uniparental inheritance, Signal peptide, Transmembrane domain, Subcellular localization, Mitochondria, Hermaphroditism

## Abstract

**Background:**

Doubly uniparental inheritance (DUI) of mitochondrial DNA in bivalves is a fascinating exception to strictly maternal inheritance as practiced by all other animals. Recent work on DUI suggests that there may be unique regions of the mitochondrial genomes that play a role in sex determination and/or sexual development in freshwater mussels (order Unionoida). In this study, one complete mitochondrial genome of the hermaphroditic swan mussel, *Anodonta cygnea*, is sequenced and compared to the complete mitochondrial genome of the gonochoric duck mussel, *Anodonta anatina*. An *in silico* assessment of novel proteins found within freshwater bivalve species (known as F-, H-, and M-open reading frames or ORFs) is conducted, with special attention to putative transmembrane domains (TMs), signal peptides (SPs), signal cleavage sites (SCS), subcellular localization, and potential control regions. Characteristics of TMs are also examined across freshwater mussel lineages.

**Results:**

*In silico* analyses suggests the presence of SPs and SCSs and provides some insight into possible function(s) of these novel ORFs. The assessed confidence in these structures and functions was highly variable, possibly due to the novelty of these proteins. The number and topology of putative TMs appear to be maintained among both F- and H-ORFs, however, this is not the case for M-ORFs. There does not appear to be a typical control region in H-type mitochondrial DNA, especially given the loss of tandem repeats in unassigned regions when compared to F-type mtDNA.

**Conclusion:**

*In silico* analyses provides a useful tool to discover patterns in DUI and to navigate further *in situ* analyses related to DUI in freshwater mussels. *In situ* analysis will be necessary to further explore the intracellular localizations and possible role of these open reading frames in the process of sex determination in freshwater mussel.

**Electronic supplementary material:**

The online version of this article (10.1186/s12864-018-4583-3) contains supplementary material, which is available to authorized users.

## Background

The order Unionoida is composed of freshwater mussels (FWM) that inhabit rivers, lakes and ponds [1]. Many gonochoric FWMs (i.e. species with separate male and female sexes), along with a few other bivalve orders (i.e. Mytiloid marine mussels, Nuculanoid nut shells, and Veneroid marine clams), do not exhibit strictly maternal inheritance of mitochondrial DNA (mtDNA) like all other animal species [2]. In contrast, these bivalves transmit mtDNA via an unusual system called doubly uniparental inheritance or DUI (for reviews see [3–5]). Under DUI, female offspring inherit maternal mtDNA (called female-transmitted or F-type mtDNA), while male offspring inherit both maternal mtDNA and paternal mtDNA (called male-transmitted or M-type mtDNA). Consequently, with respect to mitotype, males are heteroplasmic and females are homoplasmic [6]. In male offspring, F-type mtDNA is dominant throughout their somatic tissue, whereas M-type mtDNA is exclusive within gametic cells [7–9]. The respective M-type and F-type mtDNAs are also unusual, in contrast to typical animal mtDNA gene content, as each of the sex-associated mitochondrial genomes contains their own novel open reading frames (ORFs; [10]). These genes are referred to as the *m-orf* and *f-orf* in the male and female mitochondrial genomes, respectively, and these two regions code for proteins that do not show significant similarity to each other or obvious homology to any other known proteins ([10]; but also see [11, 12] who suggested that a duplicated and diverged *atp8* gene evolved into the *m-orf* gene in freshwater mussels). Immunostaining previously localized the F-ORF protein in not only the mitochondria of mature egg cells, but also on the nuclear membrane and in the nucleoplasm of the FWM *Venustaconcha ellipsiformis* (Bivalvia: Unionidae; [13]). The movement of the F-ORF protein from the mitochondria to the nucleus infers a function outside of the primary mitochondrial function of oxidative phosphorylation [13]. This finding, along with the transition of sexual strategies (detailed below) accompanied by a loss of the *m-orf*, suggests a link between sex determination and these novel ORFs in species exhibiting DUI [13]. The *in silico* analyses presented here continues the exploration of this potential link through locating putative transmembrane domains (TMs), signal peptides (SPs) and signal cleavage sites (SCSs). These molecular structures or signals may be key components of the pathway and/or mechanisms used to export these novel mitochondrial proteins to the nucleus where they could play a role in sex determination.

Recent work has shown that hermaphroditic FWMs do not possess M-type mtDNA [6, 13]. These hermaphroditic species have reverted to uniparental inheritance and transmit only an F-like mitochondrial genome, referred to as the hermaphroditic or H-type genome [13]. In several hermaphroditic species, the H-type mtDNA carries a highly modified *f-orf* gene termed the *h-orf* [13]. Because hermaphroditism has evolved independently multiple times in FWMs, *h-orfs* from different genera are not monophyletic [6, 13]. Breton et al. [13] identified four independent transitions from a gonochoric to hermaphroditic sexual strategy in the genera *Lasmigona*, *Margaritifera*, *Toxolasma* and *Utterbackia*. Interspecific comparisons of hermaphroditic FWM species showed that these *h-orfs* have highly divergent nucleotide sequences and variable amino acid hydrophobicity profiles, whereas *f-orfs* are more conserved in both respects [6, 13]. Sequence analysis of the *f-orfs* and closely related *h-orfs* suggests that the molecular modifications of *h-orfs* typically include: (1) a lengthening of the sequence and (2) the introduction of repeating sequence motifs of varying lengths [13]. Based on these characteristics, it has been hypothesized that the *f-orf* and *m-orf* genes could be key elements of the sex determination pathway in gonochoric freshwater mussels with DUI, and that associated with the shift to hermaphroditism, the *h-orf* experiences relaxed purifying selection and a change (or loss) of function compared to the *f-orf* [13].

Given the unique nature of DUI, there is considerable interest in understanding the structure and function of the various elements present in bivalve F-, M- and H-type mitochondrial genomes. This includes characterizing the *orfs* and various unassigned regions (URs), and identifying putative control regions [4, 14, 15]. Such studies are important for a better understanding of sexual strategy transitions among FWMs and to gain insights into the possible functions of the novel protein-coding genes in bivalves with DUI. In this study, we present the complete mtDNA sequence of a fifth, independently evolved hermaphroditic FWM lineage in the genus *Anodonta* sampled from Lake Konstanz, Germany. One objective is to characterize potential control regions and URs of this *A. cygnea* H-type mitochondrial genome. It has been previously documented that the F- and M-type mtDNAs of FWMs with DUI share three sizable regions of non-coding DNA including the putative *nad5–trnQ* control region, as well as two areas flanking *nad3* termed *trnF–nad3* and *nad3–trnH + A* in F-type mtDNA [10]. However, the putative control region or control regions of H-type mtDNAs have not been characterized. This is explored in the present study using data from Breton et al. [10] and the novel H-type mtDNA sequence presented herein. In addition, *in silico* analyses of H-ORFs, F-ORFs, and M-ORFs are performed to further explore the transition from gonochorism to hermaphroditism in FWMs with a focus on the topology of putative proteins associated with the *orfs* and the potential subcellular localizations and/or transport pathways of these proteins within the cell. Previous research has investigated the presence of TMs and SPs in these sex-specific ORFs; herein we extend these studies with the inclusion of additional FWM taxa and additional analyses to investigate the specific presence of SCSs and the topology of putative TMs.

## Methods

### Sequencing the complete mitochondrial genome of *Anodonta cygnea*

*Anodonta cygnea* samples were obtained from the German shore of Lake Konstanz and stored in AllProtect® (QIAGEN™). Genomic DNA was extracted using a modified saturated NaCl protocol [8, 16, 17]. Initial PCRs with modified Folmer primers [18] were conducted to sequence *cox1* (LC022me2 5’-GGTCAACAAAYCATAARGATATTGG-3′ and HC0700dy2 5’-TCAGGGTGACCAAAAAAYCA-3′ [19, 20]). Primers developed by S. Palumbi were used to amplify *rrnl* (i.e. 16 SBr 5’-CCGGTCTGAACTCAGATCACGT-3′ and 16 Sar 5’-CGCCTGTTTATCAAAAACAT-3′ [21]). Long-range polymerase chain reactions (LR-PCRs) were used to amplify two large segments of the mitochondrial genome (approximately 6 kb and 9 kb) using species-specific primers designed using Primer3 Version 4.0.0 [22] (Table 1).

**Table 1:** Species specific primers used to amplify mtDNA of *Anodonta cygnea* using LR-PCR. FOR = forward, REV = reverse, Tm = melting temperature

Primer Name	Sequence 5′ – 3′	Tm (°C)	Target Gene
ACP3 short FOR	CCAGCTAAAACAGGCAAAGC	63.7	*cox1*
ACP3 short REV	CGGGGTCTTTTCGTCTACCT	64.2	*rrnl*
ACP3 long FOR	CCTCGATGTTGGCTTAAGGA	64.0	*rrnl*
ACP3 long REV	GGAGGTTATTGGGGGATGAT	63.6	*cox1*

LR-PCRs were conducted using a Phusion® High-Fidelity DNA Polymerase Kit (New England BioLabs Inc.). Thermocycling conditions were as follows: activation at 98°C for 30 s followed by 35 cycles of 98°C for 15 s, 64°C for 40 s, and 72°C for 7 min, and a final extension at 72°C for 10 min. LR-PCR products were sequenced using PacBio RSII sequencing technology and reads were assembled with de novo assembly [23] at the McGill University and Génome Québec Innovation Centre, Montréal, Canada. The resulting circular mtDNA genome (GenBank accession MF781083) was annotated for protein-coding and RNA genes using MITOS WebServer revision 917 [24] and validated using MFannot [25]. Putative tRNA genes were further confirmed using tRNAScan-SE 2.0 [26]. The gene order of the mitochondrial genome was visualized using Geneious version 9.1.7 [27]. Images of tRNA secondary structures were generated by the MITOS WebServer revision 917 [24].

### Determining potential control regions of *Anodonta cygnea* mtDNA

URs were assessed for characteristics of control regions as follows. Putative secondary structures were identified using the mfold webserver [28] and results were visualized using VARNA version 3.93 [29]. URs were run through REPFIND Webserver 4.09 [30] to check for tandem repeats. A-T nucleotide content was checked with Geneious [27]. A comparison of *A. cygnea* URs with those of the closely-related gonochoric species *A. anatina*, as well as with the URs of the gonochoric species *Utterbackia peninsularis* and the hermaphroditic species *U. imbecillis* was conducted in Geneious to calculate sequence similarity. Sequence similarity of the UR *nad5-trnQ* was determined between H-type and closely related F-type mitochondrial genomes by aligning H-type and F-type pairs in Geneious [27] using a ClustalW plugin and default parameters.

### Assessment of a putative translocation of a portion of *nad5*

As will be detailed below, our analysis of URs in *A. cygnea* suggested an unusual translocation of a portion of the *nad5* gene. To determine if the translocated segment corresponded to a generally less conserved region of the gene, we quantified the degree of sequence conservation along the protein by (1) aligning NAD5 amino acid sequences for *A. anatina* to the amino acid sequences of two distantly-related bivalves (*Modiolus modiolus* [GenBank accession: KX821782] and *Meretrix lusoria* [GenBank accession: ACV92129.1]), and (2) by aligning this same *A. anatina* sequence to two other species for which NAD5 sequence is well-annotated and structurally well-resolved (*Homo sapiens* [UniProtKB code: P03915] and *Bos taurus* [Protein Data Bank (PDB) ID: 5LDW Chain L]). Geneious [27] was used to: (1) generate the alignment of amino acid sequences using ClustalW and a BLOSUM cost matrix, (2) annotate the region corresponding to the *nad5* translocation in *A. cygnea*, and (3) create subsequent sliding window graphs (window size = 50 bp) of the above sequences’ shared identity values. Note that in Geneious, shared identity values are determined by calculating the number of pairwise combinations that match out of the total number of possible pairwise combinations in the alignment [27]. Regarding NAD5, we attempted to determine if the translocated region corresponds to a segment outside of this protein’s main active site. Geneious [27] was used to map the putative translocated NAD5 segment onto the tertiary/quaternary structure of the Respiratory Complex 1 for *Bos taurus*. The 3D structure of Respiratory Complex 1 was retrieved in Geneious using its PDB ID: 5LDW (data based on the structures reported by [31]). To ensure that the amino acid chain within Respiratory Complex 1 that corresponds to NAD5 was correctly selected for analysis, sequence for NAD5 from *A. anatina* was aligned to protein sequence in the RCSB PDB using BLAST [32–34]. This protein BLAST analysis also served as a complementary test for our annotation of NAD5. NAD5’s protein features were assigned in our figures using the annotations reported for *H. sapiens* NAD5 (UniProtKB code: P03915) at the website InterPro (an EMBL-EBI online tool, [35]).

The presence of a translocated portion of the *nad5* gene was also tested through PCR in two additional *A. cygnea* individuals from the same population. PCRs were run using Platinum® Blue PCR SuperMix with primers Tnad5FOR1 (5-TCCATTCATCCCAAATCTAAATGC-3) and Tnad5REV1 (5- AGAGTATGGGTTGATGTAAATGGGT-3), which flank the proposed translocated region. Thermocycling conditions were: activation at 94°C for 4 min followed by 39 cycles of 94°C for 1 min, 50°C for 1 min and 72°C for 1 min, and a final extension at 72°C for 6 min. PCR products were sequenced using Sanger sequencing technology at the McGill University and Génome Québec Innovation Centre, Montréal, Canada. Sequencing results were aligned using the Clustal Omega webserver [36].

*Nad5* sequences were extracted from 12 complete mitochondrial genomes of freshwater mussels [see Additional file 1 for a list of sequence accession numbers]. All 12 *nad5* were mapped to the putative translocated *nad5* portion of *A. cygnea* and extracted in Geneious [27] using the Map to Reference function with the “highest sensitivity” parameter. An alignment of all 13 complete *nad5*, and an alignment of all extracted sequences that were mapped to the translocated portion was conducted in MEGA7 [37] to produce two separate alignment files. Each alignment file was tested for purifying selection, positive selection and strict neutrality using MEGA7 [37] with the Kumar method (available within the MEGA7 package; [37]) with 1000 bootstraps and pairwise deletion.

### *In silico* analysis of H-ORF, F-ORF and M-ORF sequences

H-ORFs were assessed for SPs using the TargetP 1.1 [38] server with default settings. If a putative SP was detected using TargetP, then the SignalP 4.1 server [39] was used to further characterize the validity of the potential SP using the “sensitive” D-cutoff value. As an alternative strategy for maximizing the detection of SPs, H-ORFs were run through SignalP a second time with the method set to exclude the possibility of TMs. Subcellular localizations (e.g. cell membrane, cytoplasm, nucleus, etc.) of H-ORFs were predicted using iLoc-Animal [40] and Euk-mPLoc 2.0 (Cell-PLoc 2.0 package) [41]. M-ORFs and F-ORFs were also assessed for SPs and subcellular localization. Finally, all ORFs were assessed for signal cleavage sites (SCS) using Geneious [27] and TargetP [38]. For comparative purposes, an alignment of an *A. anatina* F-ORF (GenBank Accession YP008802631) and an *A. cygnea* H-ORF was run and visualized in Geneious using default parameters (i.e. a BLOSUM cost matrix, a gap open cost of 10 and a gap extend cost of 0.1). Hydrophobicity plots of these ORFs were also produced using a Kyte-Doolittle scale [42]. An additional file provides a list of all GenBank accession numbers for the F-, H-, and M-ORFs used in this study [see Additional file 2].

### Mapping of predicted transmembrane domain properties on phylogenetic trees of the F-types, H-types and M-types

For the purpose of mapping properties of the predicted TMs of the F-type, H-type and M-type open reading frames in an evolutionary context, phylogenetic trees were produced based on *cox1* sequences of FWMs obtained from GenBank. An addititional file provides a list of all GenBank accession numbers for these data [see Additional file 3]. Basing these trees on *cox1* sequence data (rather than on complete genomes or multiple genes) allowed us to maximize the number of F-, H- and M-ORF sequences that could be included in the analysis (*cox1* sequence data is readily available for many FWMs). F- and H-type *cox1* sequences were aligned using ClustalW [43] with default parameters (i.e. International Union of Biochemistry cost matrix, a gap open cost of 15 and a gap extend cost of 6.66). M-type sequences were aligned separately in the same way. A Tamura-Nei model of nucleotide substitution [44] was used as per the recommendation from the model test algorithm implemented in MEGA 7.0.16 [37]. Separate Bayesian inference (BI) F-/H-type and M-type trees, respectively, were produced using BEAUti and BEAST version 2.4.6 [45] with a Yule speciation process [46] and 100 million Markov chain Monte Carlo (MCMC) steps [47] with samples taken every 1000 steps. A burn in of 10% was performed on the resulting trees. Tree samples were visualized using DensiTree [48] included with BEAST version 2.4.6 package [45], and compiled into one “best” topology using TreeAnnotator version 1.4 [49] and viewed using FigTree version 1.4.3 [50]. TM domain predictions for the F-, H- and M-ORFs were run using the transmembrane Hidden Markov Model (TMHMM) version 0.9 in a Geneious plugin and using the TMHMM Server version 2.0 [51]. These programs determine potential TM locations, number of potential TMs, and their orientation through the membrane (i.e. topology). Mesquite 3.2 [52] was used to assess the ancestral state and most parsimonious reconstruction of transitions for characters such as different TM topology and numbers of TMs present in an ORF.

## Results

### General features of the mtDNA of the hermaphroditic swan mussel, *Anodonta cygnea*

**Fig. 1:**
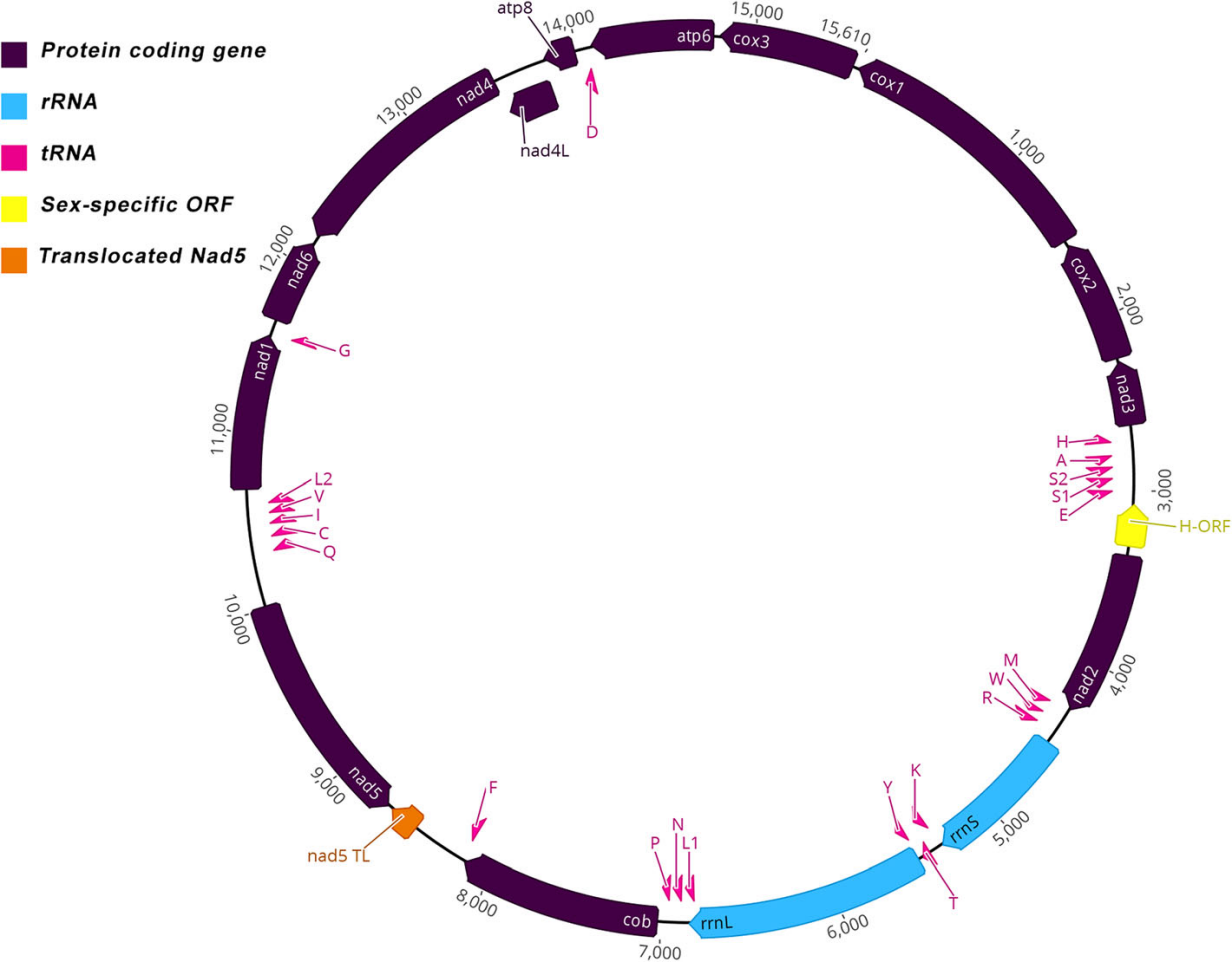
Gene order and relative size of hermaphroditic freshwater mussel (order Unionoida) *Anodonta cygnea* H-type mitochondrial genome, with annotated tRNAs and rRNAs. Image generated in Geneious [22]

The mtDNA of *Anodonta cygnea* contains the 13 protein-coding genes, and the two rRNA and 22 tRNA genes typically found in FWM mtDNAs (Fig. 1) and other animal mtDNAs [53]. This genome has the same gene order as the F-type of *A. anatina*. Similar to *A. anatina* [54], both *trnS1* and *trnS2* are missing a dihydrouridine arm [see Additional file 4], which is also the case in other FWMs (e.g. *Lampsilis ornata*) [55] and members of the order Mytiloida [56]. A fourteenth putative gene, a proposed *h-orf*, is located between *nad2* and *trnE*, the same location as the *f-orf* in *A. anatina*. At 15,607 bp, the size of the genome is typical of FWMs, and there are no obvious large duplication events. However, the putative *h-orf* of *A. cygnea* does not possess the features associated with most *h-orfs* described by Breton et al. [13]. For example, the putative *A. cygnea* H-ORF*,* at 34 amino acids, is shorter than most H-ORFs (which have a mean of 161.8, *n* = 5). It also does not possess any repetitive DNA sequence motifs as found in several other *h-orfs*. The hydrophobicity profile of the H-ORF is not strongly similar to that of the F-ORF of *A. anatina* [57], which is a previously identified general characteristic of H-ORF/F-ORF comparisons [13]. An additional file shows the hydrophobicity plots of these ORFs [see Additional file 5].

### Characterization of URs in *A. cygnea* and other H-type mtDNAs

**Fig. 2:**
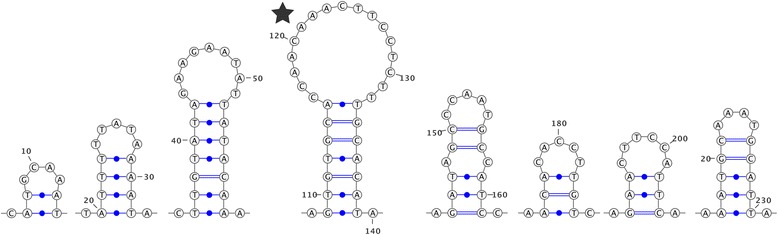
Example excerpt sequence from a potential control region of *Anodonta cygnea* within a non-coding segment between *trnF* and *nad5*. Potential secondary structures are shown, a star indicates a potential origin of replication based on structural similarities to the classic origin of replication in metazoan control regions. Note that not all secondary structures within this region are pictured. Image generated in VARNA [24]

All available H-type mitochondrial genomes shared the same three principal URs found in other FWM F-type mitochondrial genomes [10], these are outlined in Table 2. No “H-specific URs” have been found in H-type mtDNAs compared to F-type mtDNAs. Based on an assessment of all shared URs in H-type mtDNAs (Table 2), the *trnF–nad5* region is less likely to be the main control region due to its small size; however, in all instances this region does exhibit putative secondary structures. H-type URs rarely contain repeat units, but do tend to have high A-T content. The percent sequence divergence between the F-type and M-type mtDNAs proposed control region (i.e. the region between *nad5–trnQ*) [10] ranges from 43 to 50%. The percent sequence divergence between *nad5-trnQ* of F-type mitochondrial genomes and an available closely related F-type is 48–66% [Additional file 6]. The *trnF–nad3* region of *A. cygnea* possesses several potential secondary structures including one with strong similarity to a typical metazoan mitochondrial origin of replication (Fig. 2) [10].

**Table 2:** Assessment of hermaphroditic (H-type) Unionoid URs for presence of typical control region properties

Species	UR	Length (bp)	A-T content (%)	No. of repeats units	Potential Secondary structures
*Anodonta cygnea*	*nad3–trnH + trnA*	153	80.3	0	Stem loop and hairpin structure
	*nad5–trnQ*	291	66.5	0	Stem loop
	*trnF–nad5*	442	74.9	0	Stem loop and hairpin structure
*Lasmigona compressa*	*nad3–trnH + trnA*	195	79.0	0	Stem loop
	*nad5–trnQ*	208	74.0	1	Stem loop and hairpin structure
	*trnF–nad5*	55	80.0	0	Stem loop and hairpin structure
*Margaritifera falcata*	*nad3–trnH + trnA*	250	67.2	0	Stem loop and hairpin structure
	*nad5–trnQ*	171	77.2	0	Stem loop and hairpin structure
	*trnF–nad5*	47	83.0	0	Stem loop
*Toxolasma parvus*	*nad3–trnH + trnA*	300	65.3	0	Stem loop and hairpin structure
	*nad5–trnQ*	301	61.1	0	Stem loop and hairpin structure
	*trnF–nad5*	19	89.5	0	Stem loop
*Utterbackia imbecillis*	*nad3–trnH + trnA*	285	83.2	1	Stem loop
	*nad5–trnQ*	152	76.3	1	Stem loop
	*trnF–nad5*	33	90.9	0	Stem loop

An ORF was identified in the *trnF–nad5* UR of *A. cygnea* for which a BLAST search [32, 58] was conducted using both the nucleotide and putative amino acid sequences. The nucleotide search had one alignment hit with 93% identity and 35% coverage of the query sequence matching *U. peninsularis* (HM856636) within its *nad5* gene (E-value = 6 × 10^− 16^). The protein search had 49 alignment hits, with *Lasmigona compressa* (another hermaphroditic FWM) being the top hit with 100% query coverage, and a 68% identity similarity (E-value = 6 × 10^− 16^). Among the 49 hits, query coverage ranged from 90 to 100% and the percent identity ranged from 46 to 68%. These results indicate that the identified ORF is likely derived from the *nad5* gene. As such, the annotated *nad5* from *A. cygnea* and *A. anatina* were compared to elucidate how the ORF is related to the *nad5* gene. An alignment of NAD5 amino acid sequences from *A. cygnea* and *A. anatina* indicates low sequence similarity beyond residue number 420 [for the alignment, see Additional file 7a]. NAD5 in *A. cygnea* is 448 amino acids in length, whereas that of *A. anatina* is 578 amino acids long. Alignment of the putative translocated portion of NAD5 from *A. cygnea* shows that it is most similar to residue 452 to 513 of the complete *nad5 gene* from *A. anatina* [see Additional file 7b]. The predicted ORF protein from the *A. cygnea trnF–nad5* UR has a percent identity of 61.3% when aligned with NAD5 of *A. anatina*. At 1344 bp, the putative *nad5* gene of *A. cygnea* as annotated by Geneious is considerably smaller than other previously annotated FWM *nad5* genes [54, 55, 59–62]. The implication of the potential translocation of a part of *nad5* within the *A. cygnea* mtDNA is addressed below.

### *Anodonta cygnea* may have a translocated portion of *nad5*

**Fig. 3:**
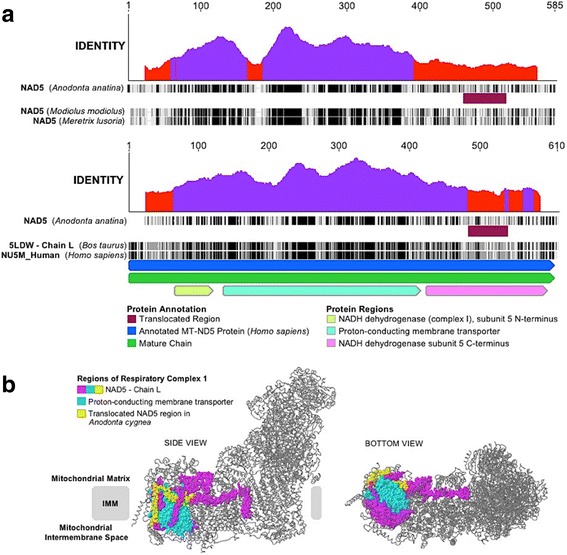
Analysis of translocated *nad5* region in *Anodonta cygnea*. **a** Upper plot shows NAD5 protein alignment for members of family Unionoida (*Anodonta anatina*; KF030964), Mytiloida (*Modiolus modiolus*; KX821782) and Veneroida (*Meretrix lusoria*; ACV92129). Lower plot shows the same *A. anatina* sequence aligned to *Homo sapiens* and *Bos taurus* NAD5 sequences; UniProtKB code P03915 and PDB ID: 5LDW Chain L, respectively. In both upper and lower plots the region corresponding to the partial *nad5* translocation in *A. cygnea* is annotated. Functional regions are coloured according to the legend and descriptions provided in InterPro for *H. sapiens* NAD5 (accession P03915). Amino acids in alignments are colour coded by similarity (black = 100% similar, dark gray = 80–100% similar, light grey = 60–80% similar, white = < 60% similar). Identity graphs were generated with a sliding-window size of 50 bp, furthermore red and purple colours along plots represent positions that have very low identities (30%), and less than complete identities (30% to < 100%), respectively. See methods for an explanation of how similarity was calculated. Images were generated in Geneious [22]. **b** Partial *nad5* translocation in *A. cygnea* relative to the 3-dimentional structure of Respiratory Complex 1 (PDB ID: 5LDW). In particular, NAD5 (amino acid Chain L) is coloured. Note that structures are based on work in [26]

Given that it appears that an internal region of *nad5* has been translocated in *A. cygnea*, we further assessed whether the translocated sequence represents a portion of NAD5 that is generally less conserved, or if the translocated region contains any portion of the active site of the protein. It appears likely that the translocated portion of NAD5 is not under strong selection as the nucleotide sequence of this portion within other FWM species ranges from only an interspecific percent identity of 45.2–56.6%, except in the unusual case of *L. compressa* (another hermaphrodite), which is 83% similar to this portion in *A. cygnea*. BLAST searches of this stretch of NAD5 with other FWM species resulted in similar percent identities among species, whereas the percent identity of NAD5 overall (including this region) ranged from 81.7–88.6% among the same set of FWM species. A multiple sequence alignment of bivalve NAD5 amino acids showing both a sliding window of identity along with the approximate location of the translocated NAD5 portion annotated, also suggests that this region is not especially well conserved within bivalves (Fig. 3a [upper plot]). Furthermore, when this analysis is expanded to include a comparison to two other metazoans outside Bivalvia for which NAD5’s protein structure and functional regions have been more thoroughly classified/annotated (e.g. *Homo sapiens* and *Bos taurus*), through these comparisons we conclude that the translocated region is from a less conserved portion of the gene (Fig. 3a [lower plot]). When the translocated segment is mapped onto the structure of NAD5 (also known as “Chain L”, and which was found to be *A. cygnea*’s closest RCSB protein data bank alignment hit to an amino acid chain within the *B. taurus* Respiratory Complex 1 structure [E-value = 9.29 × 10^− 68^]) [32], it is clear that the segment corresponds to a portion of NAD5 that is outside the protein’s main proton-conducting membrane transporter region (Fig. 3b) [31, 35].

Finally, we tested strict-neutrality of both the (a) complete *nad5* from several FWM species [see Additional file 1 for the list of species used with accession numbers], and the (b) corresponding region of *nad5* that may be translocated in *A. cygnea* to determine whether this region of *nad5* is less conserved (Table 3). Strict neutrality was rejected (H_o_: d_n_ = d_s_; two tailed test) with a *p*-value of < 0.001 in both cases (test statistic = − 14.789 and 6.493, for (a) and (b) respectively). Tests of purifying and positive selection (both one-tailed) were conducted, based on rejecting strict-neutrality, on both the complete (a) and (b) (Table 3). These results suggest that overall *nad5* from FWM species is under purifying selection, whereas the region corresponding to the putative translocated portion of *nad5* found in *A. cygnea* alone is under positive selection.

**Table 3:** Z-test of selection results for purifying selection (H_o_: d_n_ = d_s_, H_a_: d_n_ < d_s_) and positive selection (H_o_: d_n_ = d_s_, H_a_: d_n_ > d_s_) on alignments of **(A)** complete *nad5* from 12 species with 543 positions, and **(B)** *nad5* sequences corresponding to a putative translocated portion of *nad5* from *Anodonta cygnea* (MF781083) from 12 species with 45 positions in the analysis. Alignments by ClustalW [36] and Z-tests were conducted in MEGA7 [37] using the Kumar method, 1000 bootstraps, and pairwise deletion. Statistically significant results are highlighted in bold using α = 0.05

A			B		
Test	P-value	Test statistic	Test	P-value	Test statistic
Purifying selection	**< 0.001**	14.932	Purifying selection	1.000	−6.456
Positive selection	1.000	−14.495	Positive selection	**< 0.001**	6.842

Given these results, we suggest that the area of *nad5* that is translocated in *A. cygnea* is likely a relatively unconstrained part of the *nad5* gene. This putative translocated portion of *nad5* was also present in all three samples of *A. cygnea* tested (including the individual used for sequencing the complete mtDNA) [see Additional file 8], suggesting that it is a real phenomenon and not a result of a PCR or sequencing artifact.

### *In silico* analysis of H-ORFs compared to F-ORFs

The amino acid sequence length of H-ORFs varies considerably with the smallest reported to date being for *A. cygnea* at 34 amino acids long and the largest for *U. imbecillis* with 286 amino acids. All subsequent analyses of H-ORFs in particular are inevitably going to be affected by this variation in length. Presence of a TM were predicted for all H-ORFs were at 100%, with the exception of *A. cygnea* (TMHMM output at < 60%); however, we did not exclude the possibility of a TM in *A. cygnea* because of its relatively short length. The topology for all TM predictions included a cytoplasmic to extracellular (C-TM-E) crossing of the membrane, although this orientation was lower for *A. cygnea* (63%) and particularly low for *M. falcata* (22%). TargetP detected an SP in all H-ORFs (Reliability class (RC) = 1 for *A. cygnea*, *L. compressa,* and *L. subviridus*; RC = 4 for *M. falcata*, and *T. parvus*; RC = 5 for *U. imbecillis*), and putatively assigned all H-ORF SPs as part of a secretory pathway except for *M. falcata*, which it identified as a mitochondrial SP (note: even for the lowest RC value of 5, the probability of an SP being present in a sequence is greater than would be expected from a random sequence [39]). With SignalP parameters set to “sensitive” and with a potential TM presence, only *L. subviridis* came back positive for an SP. When SignalP parameters were set to exclude the possibility of a TM being present, all H-ORFs came back as positive for an SP except *M. falcata*. TMHMM webserver predicted an SP in all H-ORFs (6 total) and F-ORFs (34 total), except for the F-ORFs of *C. monodonta* and *U. peninsularis*. A SCS was predicted in 30 of 36 F-ORFs and 4 of 6 H-ORFs.

Subcellular localization analysis predicted the following: (1) that the *T. parvus* H-ORF was localized to the cytoplasm and nucleus (iLoc-Animal) or extracellular and nucleus (EuK-mPLoc), (2) that the *L. compressa* H-ORF was localized to nucleus (iLoc-Animal, EuK-mPLoc), (3) that the *L. subviridis* H-ORF was localized to cellular membrane and plasma membrane (iLoc-Animal) or extracellular and nucleus (EuK-mPLoc), (4) that the *M. falcata* H-ORF was localized to cellular membrane and plasma membrane (iLoc-Animal) or extracellular (EuK-mPLoc), (5) and finally, that the *U. imbecillis* H-ORF was localized to cytoplasm (iLoc-Animal) or cellular membrane (EuK-mPLoc). EuK-mPLoc could not assess the potential localization of the *A. cygnea* H-ORF due to its small size, and iLoc-Animal did not localize it to any area, possibly also because of its small size. While H-ORFs overall did not have a clear subcellular localization, the F-ORF analysis using EuK-mPLoc predicted that all F-ORFs, except for *C. monodonta* and *U. peninsularis*, have an extracellular localization. Some other hits among the F-ORFs included localization to the cytoplasm, nucleus, and mitochondria; in addition, *U. peninsularis* exhibited a unique localization to endoplasmic reticulum and cell membrane. To summarize, the localization of the ORF proteins, and particularly the H-ORFs, as determined by *in silico* analyses is highly variable. It must be noted, however, that with the exception of the H-ORFs present in the two species of *Lasmigona*, each of these proteins represent an independent transition from an F-ORF in a gonochoric species, to an H-ORF in a hermaphroditic species. The possible implications of the independent nature of these multiple transitions to the hermaphroditic state will be discussed below.

### *In silico* analysis of M-ORFs

All 11 M-ORFs contained at least one potential TM with variable topologies. All M-ORFs possessed an SCS predicted by either or both Geneious and TargetP. All predicted SCS were within the potential TM hydrophobic stretch of amino acids. No clear pattern of subcellular localization hits of M-ORFs resulted from the EuK-mPLoc or iLoc-Animal analyses.

### Determination of evolutionary pattern(s) of TM predictions and their associated topologies among the sex-associated mitochondrial ORF proteins

**Fig. 4:**
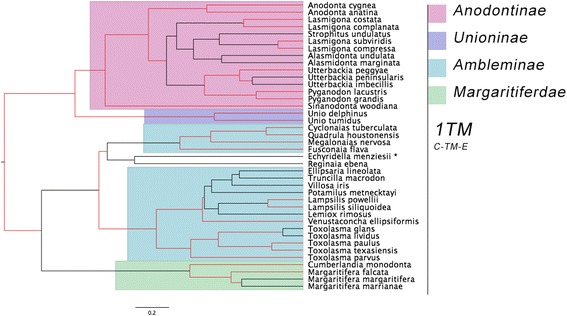
F-type and H-type BI phylogenetic tree based on *cox1* DNA sequences. Posterior probabilities > 0.75 are marked in red. A (*) denotes a species that has potential of possessing two TMs. Number of predicted transmembrane domains (TM) and their topology are shown (E = extracellular, C = cytoplasmic). Subfamilies are colour-coded. A single species represents subfamily Hyriidae (*Echyridella menziesii*), and Ambleminae (*Reginaia ebena*). Tree annotated in FigTree [45]

**Fig. 5:**
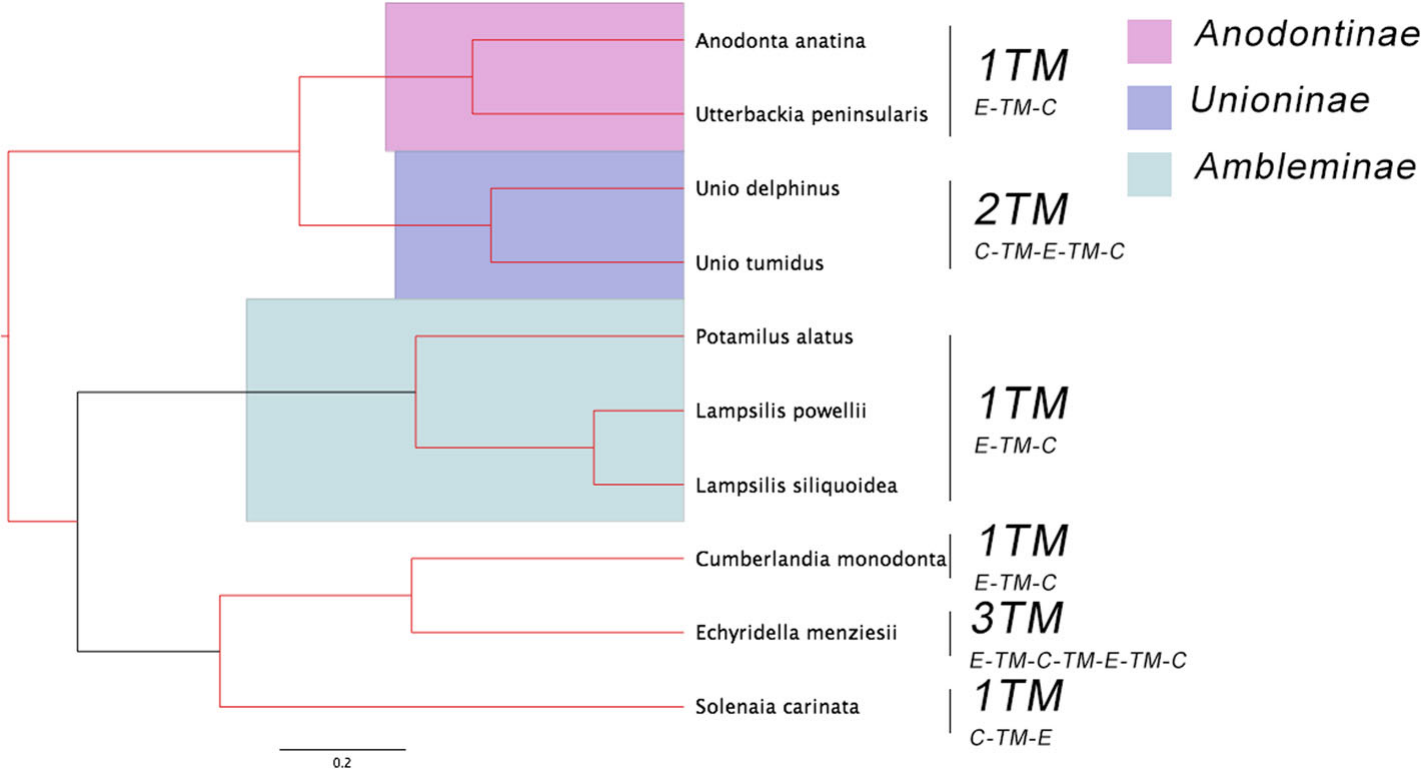
M-type BI phylogenetic tree of based on *cox1* DNA sequences. Posterior probabilities > 0.75 are marked in red. Number of predicted transmembrane domains (TM) and their topology are shown (E = extracellular, C = cytoplasmic). Subfamilies are colour-coded. A single species represents subfamily Margaritiferdae (*Cumberlandia monodonta*), Hyriidae (*Echyridella menziesii*), and Gonideinae (*Solenaia carinata*). Tree annotated in FigTree [45]

The following results are based on constructing BI trees of F-/H-type or M-type *cox1* sequences and examining the trees for evolutionary patterns of F-, H- and M-ORF TM predictions and topologies as identified in the previous section. Almost all gonochoric and hermaphroditic species have one F-/H-ORF TM prediction with a C-TM-E topology (Fig. 4), although some predictions are not at 100% likelihood for either orientation or presence of a TM (< 80% likelihood). The only major exception was the F-ORF of *Ellipsaria lineolata*, which potentially possesses two TMs, but the topology of these TMs was not strongly supported (correct orientation of the first TM at only 8% probability). There is no clear link between phylogenetic placement on the *cox1* tree and the absence of an SCS predicted within the F-ORFs, except for the genus *Toxolasma*. A parsimonious reconstruction of both number of TMs and their topologies suggests that one TM and an extracellular-TM-cellular (E-TM-C) topology is the ancestral state of all M-type FWMs included (Fig. 5).

## Discussion

The complete mtDNA genome of *Anodonta cygnea* is similar to other F-/H-type FWM mtDNA genomes with respect to location of protein coding genes, rRNAs, tRNAs and the H-ORF. An interesting exception is a translocation of a portion of *nad5*. The principal URs of *A. cygnea* and other H-type mtDNAs share some, but not all, common features of F-type URs. As expected based on prior analyses of FWM H-ORFs with F-ORFs of closely related species [13], the putative H-ORF of *A. cygnea* shows little similarity to the F-ORF of a close relative, *A. anatina* (Additional file 5: Figure S2). However, somewhat unexpectedly, it does not contain the common features of other H-ORFs as outlined by Breton et al. [13]. Consistent with other hermaphroditic FWMs, there is no evidence of an M-type mitochondrial genome in this population of hermaphroditic *A. cygnea* [6]. Further insights into the putative H-ORF of *A. cygnea*, and molecular characterization of F-/H-ORFs of FWMs in general are discussed below.

### A portion of *nad5* is translocated to the *trnF-nad5* UR in *Anodonta cygnea*

Our analysis of the *trnF–nad5* UR suggested the presence of a partial *nad5* translocation. Serb and Lydeard [55] previously used the same approach based on amino acid similarity to identify a translocated portion of *atp8* in the F-type genome of *Unio japanensis.* Although *nad5* does not appear to be highly modified among *A. anatina*, *U. peninsularis*, and *U. imbecillis,* it is possible that the region of *nad5* that is translocated is less conserved. If a portion of *nad5* can translocate (or the parts of *nad5* flanking it) then it is possible that selective pressures are relatively unequal across the gene and that oxidative phosphorylation functions are not interrupted with the movement of this piece. This piece of *nad5* could be transcribed and translated separately from the remainder of the gene given that both pieces have their own start codons. It is likely that the amino acids that form the active sites of the protein’s tertiary structure would be under stronger selective constraints than neighbouring amino acids that play a primarily structural role (e.g. [63]). Regarding this point, our additional analyses mapping the translocated segment to the tertiary/quaternary structure of Respiratory Complex 1 and conducting Z-tests of selection, further support the notion that the translocation is outside the main proton-conducting region of NAD5 and that it corresponds to a region within the protein’s C-terminus. Overall, we hypothesize that NAD5 of *A. cygnea* functions adequately with this component being translocated. Future research would require functional studies to test this hypothesis.

### Putative control region(s) appear similar among F- and H-type mtDNAs

Although secondary structures were located within all principal URs, and within the longest unassigned region of all complete H-type mtDNAs available, it is difficult to pinpoint a main control region similar to other metazoan mitochondrial genomes. The proposition that *nad5–trnQ* UR [10] is the main control region of F-type mtDNA is also possible for the H-type mtDNA; however, *nad3–trnH + trnA* also appears to be a potential candidate for the primary control region. Curiously, only three URs within five H-type complete mtDNAs (15 URs total) assessed contain repeats, which is notably different than the pattern described in Breton et al. [10]. In that study, all main URs of the seven F-type mtDNAs assessed (35 URs total) contained at least one repeat and at most 33. It is unclear why a shift from a gonochoric sexual strategy to a hermaphroditic sexual strategy would accompany a loss in repetitive sequence within URs, however, there may be something specific about repeat arrays in URs that are no longer required following this shift in reproductive strategy and the concomitant changes in processes of germ line, gonad tissue and gamete development.

Within F-type mtDNA, *nad5–trnQ* ranges from 202 to 450 bp (excluding the exceptional case of *U. japanensis* at 1196 bp), whereas this region ranges from 152 to 291 bp in H-type mitochondrial genomes. There does not appear to be a reduction in complete mtDNA length overall with the transition from gonochorism to hermaphroditism. Figure 2 provides an example of secondary structures, and thereby potential nucleotide motifs such as an origin of replication or transcription, occurring within the *A. cygnea trnF–nad3* UR. However, similar structures occur within main URs from all other H-type complete mtDNAs analyzed and this feature is therefore not unique to *A. cygnea*. As noted in Breton et al. [10], these URs are associated with a change in the direction of transcription within the mitochondrial genome, and an origin of transcription occurring within each would seem to make sense.

The transition from a gonochoric to hermaphroditic sexual strategy in FWMs must involve modifications to cellular processes to facilitate production of both types of gametes in one individual. If mitochondrial genes are involved in the developmental pathways that determine sex, then the F-, M, and H-type genomes may contain distinct genetic signatures that play a role in these processes. Examining the mitochondrial URs further will provide us with a better understanding of the basis of these developmental systems. However, based on the present analysis it appears that the only major difference in URs between F- and H-type mtDNAs is the loss of tandem repeats.

### *In silico* analyses of H-, F-, and M-ORFs

The *in silico* analyses presented here agree with the findings of Mitchell et al. [11] in that (1) TMs were predicted in almost all ORFs (although their likelihood varied) and (2) SPs were predicted in almost all ORFs, but again these predictions remains somewhat less certain as “sensitive” parameters were used for this analysis. SPs possess a tripartite structure that includes a hydrophilic region, a hydrophobic region and an SCS [64]. The hydrophilic region usually consists of positively charged amino acids, varies in length [65], and generally has the highest variability of all three regions [66]. In contrast, the hydrophobic region is considered the most essential feature of an SP [67]. Although some SP predictions were uncertain, the confirmation of both a hydrophobic region and an SCS leave only the variable hydrophilic region to be confirmed; theoretically this variability (especially in these novel ORFs with a high substitution rate) could result in under-identification of the region in question. Although SPs share this tripartite structure, they do not possess a single characteristic sequence motif and instead can tolerate mutations [67, 68]. SPs target proteins to a membrane and can reach their destination through different pathways. The topology of a protein during translocation through a protein conducting channel is C-TM-E, which, interestingly, is the orientation consistently predicted for F- and H-ORFs (Fig. 4). M-ORFs adopt this orientation less often (Fig. 5). If the SCSs of SPs leave a significant hydrophobic region after cleavage then this structure could function as an anchor for the SP at its target destination [65, 69, 70]. The SCS locations across these novel ORFs vary in relation to their placement along the hydrophobic stretch of amino acids, therefore the number of hydrophobic amino acids remaining after cleavage is variable. Consequently, it is inconclusive if these proteins will be anchored or not at their final destination. These speculations must be investigated with future, targeted approaches. Interestingly, all known examples of TMs formed from the hydrophobic region of SPs have a viral origin [64]. Both Milani et al. [71] and Mitchell et al. [11] suggest that these ORFs could have a viral origin, although Mitchell et al. [11] and Guerra et al. [12] also suggest that the process of gene duplication and subsequent modification of a mitochondrial gene may have been responsible. Determining whether or not these *orfs* produce anchored proteins is relevant for testing these hypotheses regarding the origin of the *orf* genes.

For several FWMs, Mitchell et al. [11] identified motifs and domains of F-ORFs and M-ORFs related to cell membrane and surface anchoring, but independent *in situ* verification of the localization of these polypeptides would be useful. In the case of *V. ellipsiformis,* the F-ORF was associated with both cytoplasm (mitochondria and nucleoplasm) and membrane (plasma membrane), but this does not necessarily imply that these structures are the final destinations for these proteins. Multiple cleavage sites were predicted for many F-ORFs, consequently it may be possible to have both anchored and non-anchored versions of one protein. However, it should be noted that for the F-ORF of *V. ellipsiformis* only one SCS was predicted using TargetP. This SCS was located near the end of the hydrophobic stretch leaving relatively few hydrophobic amino acids to act as an anchor. It has also been suggested that SP fragments, which have been cleaved at the SCS, may have post-targeting functions and that the cleaved proteins themselves might undergo additional processing [66]. This further complicates identifying the potential function(s) of these novel ORFs given their multiple putative SPs and SCSs. To add another layer of complexity, SPs are not always cleaved, particularly in the case of some viral proteins [65]. Collectively, these factors shed some light on the variety of subcellular localization hits resulting from our *in silico* analyses. Furthermore, the putative localizations do not always agree with the results of *in situ* analyses. For example, the *V. ellipsiformis* F-ORF was localized as extracellular based on the *in silico* analysis and not predicted to occur in any of the locations identified through immunostaining [13]. The difficulty in accurately predicting functions and sub-cellular localizations, specifically, for these ORFs may be because (1) they are not homologous to any other known and previously characterized proteins, (2) they may be participating in unique functions associated with a unique phenomenon (i.e. DUI), and (3) they are produced in the mitochondria and migrating to the nucleus (also a unique process) and standard *in silico* analysis programs are not optimized to characterize such proteins. For example, the programs iLoc-Animal and EuK-mPLoc rely on the Swiss-Prot Database [40, 41, 72, 73], which is not optimized to predict localization for mitochondrial proteins that are exported to other cellular structure(s). *In silico* analyses could still be useful for constructing hypotheses that can tested using *in situ* analytical techniques such as the immunostaining conducted by Breton et al. [13]. These or similar analyses such as the electrophoretic mobility shift analyses (EMSA) conducted by Kyriakou et al. [74] to characterize interactions between proteins and mitochondrial DNA sequence motifs will be necessary to create a more complete picture of what these fascinating proteins are doing, and if they could be a component in a sex determination mechanism in bivalves possessing DUI.

### Putative TM topology of F-/H-ORF and M-ORFs exemplifies the variable selective constraints placed on F-, H- and M-type mtDNA

The F-type and H-type *cox1* tree (Fig. 4) closely reflects the groupings suggested in Lopes-Lima et al. [75], with the exception of the position of *Echyridella menziesii*. However, there is not strong support for the placement of this particular species on the tree (i.e. < 75% posterior probability). It is possible that including additional genes would resolve this tree topology to more closely reflect the BI tree presented in Breton et al. [13], which was based on both *cox1* and *nad1* sequences. The *E. lineolata* F-ORF was scored as potentially having two TMs, however, the first TM likelihood was < 80%, whereas the second TM was above this threshold based on TMHMM webserver results. The topology of the putative TM was also somewhat uncertain (8% probability of the correct topology being predicted), further reducing the likelihood of this region forming a TM. Consequently, it is likely that all the FWM species included in the F-/H-type *cox1* tree possess an ORF with a single TM domain and a C-TM-E orientation. These trees (Figs. 4 and 5) suggest that a stronger selective pressure is placed on the F-ORFs and potentially the H-ORFs to maintain a hydrophobic region that forms a TM. An analysis of a total of 40 FWM species (either female or hermaphroditic) provides a clear indication that this TM and its topology is being maintained across several subfamilies. In contrast, analysis of a total of 10 males suggest that the number of TMs and their topology is not maintained across several subfamilies, but may be maintained within some subfamilies. Interestingly, among closely related subfamilies on the M-type tree for the families Anodontinae and Unioninae (this study; [76]), a drastic change from one TM and an E-TM-C topology to two TMs and a C-TM-E-TM-C topology occurs. The ancestral topology of F-/H-ORFs is opposite to the ancestral topology predicted for M-ORFs. *M-orf* and *f-orf* are likely not homologous genes and are derived from separate evolutionary events [13, 71], it is therefore not surprising that they have different ancestral topologies. However, it is unclear if DUI or these novel ORFs appeared first as it has yet to be determined precisely what function the ORFs have, if they all have similar functions, or if some are functioning while others (i.e. H-ORFs) have lost functionality.

A comparison between these two trees exemplifies the selective constraints placed on the F-ORFs compared to the M-ORFs and similarly the selective constraints placed on F-type and M-type mtDNA of species with DUI. Although it has been shown that both FWM ORFs have a high amino acids substitution rate [10], it has also been shown that M-type mtDNA typically evolves much more quickly than F-type mtDNA in bivalves exhibiting DUI [76–78]. It is possible that selective constraints placed on F-ORFs are much higher than those placed on M-ORFs, which is illustrated by the multiple character states in this relatively small sample of FWM M-ORFs. Although patterns have emerged from the comparison of H-ORFs and closely related F-ORFs [13], H-ORF comparisons show a high level of sequence divergence and their hydrophobicity profiles vary greatly [13]. This is particularly interesting given that the hermaphroditic reproductive mode has evolved independently multiple times within order Unionoida.

## Conclusions

Overall, the transition from gonochorism to hermaphroditism appears to conserve various features of the F-type mtDNA genome during modification to the H-type mtDNA genome. This is highlighted by our assessment of URs and our *in silico* analysis of F-ORFs compared to H-ORFs. To summarize, we have (1) added a fifth complete mitochondrial genome to the existing pool of data, (2) demonstrated the first potential translocated *nad5* gene portion within a hermaphroditic FWM, (3) provided further evidence of signal peptides and evidence of SCS in the F-, and H-ORFs, which may be carrying out functions related to sex determination after transportation to the nucleus and (4) linked the number of TMs and their topology to FWM F- and M-type lineages. However, the putative TMs and potential SPs within M-ORFs remain unclear and inconsistent. Further exploration of potential SPs and the subcellular localization of these novel proteins (F-, M- and H-ORF) should include *in situ* analysis to overcome barriers associated with the novelty of these proteins within *in silico* analysis (e.g. using programs based on protein banks with no clear homology to F-, M- and H-ORFs).

## Additional files

Additional file 1: **Table S1.** Genbank accessible *nad5* sequences used in this study. (PDF 68 kb)

Additional file 2: **Table S2.** Genbank accessible F-ORFs, H-ORFs, and M-ORFs used in this study. (PDF 72 kb)

Additional file 3: **Table S3.** Genbank accessible *cox1* sequences used in this study [79–85] (PDF 81 kb)

Additional file 4: **Figure S1.** Predicted tRNA structures for all 22 tRNA of H-type mitochondrial DNA in *A. cygnea*. Left-to-right and top-to-bottom order follows their placement within the annotated complete mitochondrial genome beginning at base 2668. (PDF 164 kb)

Additional file 5: **Figure S2.** Comparative analysis of an F- and H-ORF of closely related species. (A) Kyte-Doolittle hydrophobicity plots of the *Anodonta anatina* F-ORF (GenBank Accession YP008802631) and *Anodonta cygnea* H-ORF (this study). (B) Amino acid alignment of the same *A. anatina* F-ORF and *A. cygnea* H-ORF, where black represents the same amino acid, grey is similar and white is different. (PDF 116 kb)

Additional file 6: **Table S4.** Sequence divergence of *nad5-trnQ* between H-type and closely related F-type mitochondrial DNA. (PDF 43 kb)

Additional file 7: **Figure S3.** NAD5 amino acid alignment of *Anodonta cygnea* and *Anodonta anatina*. (PDF 84 kb)

Additional file 8: **Figure S4.** Nucleotide alignment of three translocated portions of *nad5* from three *Anodonta cygnea* individuals. *A.cygnea* 3 is the subject of this paper (Genbank accession MF781083). (PDF 70 kb)

## Abbreviations

BI: Bayesian inference
C-TM-E: Cytoplasmic to extracellular
DUI: Doubly uniparental inheritance
EMSA: Electrophoretic mobility shift analyses
E-TM-C: Extracellular-TM-cellular
FWM: Freshwater mussels
LR: Long-range
mtDNA: mitochondrial DNA
ORFs: open reading frames
PCR: polymerase chain reaction
PDB: protein data bank
RC: reliability class
SCSs: signal cleavage sites
SPs: signal peptides
TMs: transmembrane domains
URs: unassigned regions
