# Supplementary material for: The complete mitochondrial genome of the hermaphroditic freshwater mussel Anodonta cygnea (Bivalvia: Unionidae): in silico analyses of sex-specific ORFs across order Unionoida
Source: BMC Genomics. 2018 Mar 27;19:221. doi: 10.1186/s12864-018-4583-3 (PMC5870820; doi:10.1186/s12864-018-4583-3)
Supplement: Supplementary file 1 — Table S1. Genbank accessible nad5 sequences used in this study. (PDF 68 kb) [file 12864_2018_4583_MOESM1_ESM.pdf]

Additional File 1. Genbank accessible complete mitochondrial genomes used in this study for the purpose of extracting complete *nad5* nucleotide sequences from freshwater mussel species.

Species	Mitotype	Accession
Anodonta anatina	F	KF030964
Anodonta arcaeformis	F	KF667530
Anodonta euscaphys	F	KP187851
Anodonta lucida	F	KF667529
Hyriopsis cumingii	F	FJ529186
Lamprotula leai	F	JQ691662
Lampsilis ornata	F	AY365193
Lampsilis powellii	F	MF326971
Lampsilis siliquoidea	F	MF326973
Pyganodon grandis	F	FJ809754
Sinanodonta woodiana	F	HQ283344
Toxolasma parvum	H	HM856639
