# Supplementary material for: The complete mitochondrial genome of the hermaphroditic freshwater mussel Anodonta cygnea (Bivalvia: Unionidae): in silico analyses of sex-specific ORFs across order Unionoida
Source: BMC Genomics. 2018 Mar 27;19:221. doi: 10.1186/s12864-018-4583-3 (PMC5870820; doi:10.1186/s12864-018-4583-3)
Supplement: Supplementary file 2 — Table S2. Genbank accessible F-ORFs, H-ORFs, and M-ORFs used in this study. (PDF 72 kb) [file 12864_2018_4583_MOESM2_ESM.pdf]

Additional File 2. Genbank accessible F-ORFs, H-ORF, and M-ORFs used in this study.

Species	Mitotype	Reference	Accession
Alasmidonta marginata	F	[13]	AEC14025
Alasmidonta undulata	F	[13]	AEC14028
Anodonta anatina	F	[54]	YP008802631
	M	[59]	AGS17944
Anodonta cygnea	H	This study	MF781083
Cumberlandia monodonta	F	[13]	AEC14034
	M	[11]	AOC83868
Cyclonaias tuberculata	H	[13]	AEC14036
Echyridella menziesii	F	[13]	AEC14042
	M	[11]	AOC83866
Ellipsaria lineolata	F	[13]	AEC14037
Fusconaia flava	F	[13]	AEC14041
Lampsilis powellii	F	Unpublished; Robicheau et al.	MF326971
	M	Unpublished; Robicheau et al.	MF326972
Lampsilis siliquoidea	F	Unpublished; Robicheau et al.	MF326973
	M	Unpublished; Robicheau et al.	MF326974
Lasmigona complanata	F	[13]	AEC14052
Lasmigona compressa	H	[13]	HM856638
Lasmigona costata	F	[13]	AEC14056
Lasmigona subviridis	H	[13]	HM856640
Lemiox rimosus	F	[13]	AEC14057
Margaritifera falcata	H	[13]	AEC14216
Margaritifera margaritifera	F	[13]	AEC14059
Margaritifera marrianae	F	[13]	AEC14061
Megalonaias nervosa	F	[13]	AEC14063
Potamilus alatus	M	[60]	AQQ08223
Potamilus metnecktayi	F	[13]	AEC14065
Pyganodon grandis	F	[13]	AEC14088
Pyganodon lacustris	F	[13]	AEC14098
Quadrula houstonesis	F	[13]	AEC14100
Reginaia ebena	F	[13]	AEC14038
Sinoanodonta woodiana	F	[62]	ADP23839
Solenaia carinata	M	[61]	AGX27820
Strophitus undulatus	F	[13]	AEC14109
Toxolasma glans	F	[13]	AEC14115
Toxolasma lividus	F	[13]	AEC14116
Toxolasma parvum	H	[11]	AOC83870
Toxolasma paulus	F	[13]	AEC14123
Toxolasma texasiensis	F	[13]	AEC14136
Truncilla macrodon	F	[13]	AEC14137
Unio delphinus	F	[79]	AMX22403
	M	[79]	AMX22424
Unio tumidus	F	[80]	AQM37870
	M	[80]	AQM37820
Utterbackia imbecillis	H	[13]	AEC14273
Utterbackia peggyae	F	[13]	AEC14165
Utterbackia peninsularis	F	[13]	ADL62609
	M	[13]	ADL62595
Venustaconcha ellipsiformis	F	[13]	AEC14189
Villosa iris	F	[13]	AEC14192
