# Supplementary material for: The complete mitochondrial genome of the hermaphroditic freshwater mussel Anodonta cygnea (Bivalvia: Unionidae): in silico analyses of sex-specific ORFs across order Unionoida
Source: BMC Genomics. 2018 Mar 27;19:221. doi: 10.1186/s12864-018-4583-3 (PMC5870820; doi:10.1186/s12864-018-4583-3)
Supplement: Supplementary file 3 — Table S3. Genbank accessible cox1 sequences used in this study [79–85] (PDF 81 kb) [file 12864_2018_4583_MOESM3_ESM.pdf]

Table S2. Genbank accessible *cox1* sequences used in this study.

Species	Mitotype	Reference	Accession
Alasmidonta marginata	F	[13]	HM849059
Alasmidonta undulata	F	[13]	HM849061
Anodonta anatina	F	[54]	EF440347
	M	[59]	KF030963
Anodonta cygnea	H	This study	
Cumberlandia monodonta	F	[13]	HM849068
	M	[12]	KU873124
Cyclonaias tuberculata	H	[81]	HM230410
Echyridella menziesii	F	[13]	HM849074
	M	[12]	KU873122
Ellipsaria lineolata	F	[13]	HM849071
Fusconaia flava	F	[81]	HM230370
Lampsilis powellii	F	Unpublished; Robicheau et al.	MF326971
	M	Unpublished; Robicheau et al.	MF326972
Lampsilis siliquoidea	F	Unpublished; Robicheau et al.	MF326973
	M	Unpublished; Robicheau et al.	MF326974
Lasmigona complanata	F	[13]	HM849078
Lasmigona compressa	H	[13]	HM849085
Lasmigona costata	F	[13]	HM849089
Lasmigona subviridis	H	[13]	HM849092
Lemiox rimosus	F	[81]	HM230406
Margaritifera falcata	H	[13]	HM849094
Margaritifera margaritifera	F	[13]	HM849098
Margaritifera marrianae	F	[13]	HM849094
Megalonaias nervosa	F	[82]	AY655007
Potamilus alatus	M	[60]	KU559010
Potamilus metnecktayi	F	[13]	HM849099
Pyganodon grandis	F	[13]	HM849107
Pyganodon lacustris	F	[13]	HM849114
Quadrula houstonesis	F	[83]	KT285649
Reginaia ebena	F	[13]	HM849072
Sinoanodonta woodiana	F	Soroka and Burzyński, unpublished	HQ283347
Solenaia carinata	M	[61]	KC848655
Strophitus undulatus	F	[13]	HM849122
Toxolasma glans	F	[13]	HM849128
Toxolasma lividus	F	[81]	JF326436
Toxolasma parvum	H	[13]	HM849154
Toxolasma paulus	F	[13]	HM849164
Toxolasma texasiensis	F	[82]	AY655023
Truncilla macrodon	F	[83]	KT285658
Unio delphinus	F	[76]	KP217927
	M	[79]	KT326918
Unio tumidus	F	[62]	AY074807
	M	[80]	KY021075
Utterbackia imbecillis	H	[13]	HM849174
Utterbackia peggyae	F	[13]	HM849191
Utterbackia peninsularis	F	[13]	HM849198
	M	[13]	HM856635
Venustaconcha ellipsiformis	F	[85]	KC537304
Villosa iris	F	[13]	HM849201
