# Supplementary material for: The complete mitochondrial genome of the hermaphroditic freshwater mussel Anodonta cygnea (Bivalvia: Unionidae): in silico analyses of sex-specific ORFs across order Unionoida
Source: BMC Genomics. 2018 Mar 27;19:221. doi: 10.1186/s12864-018-4583-3 (PMC5870820; doi:10.1186/s12864-018-4583-3)
Supplement: Supplementary file 4 — Figure S1. Predicted tRNA structures for all 22 tRNA of H-type mitochondrial DNA in A. cygnea. Left-to-right and top-to-bottom order follows their placement within the annotated complete mitochondrial genome beginning at base 2668. (PDF 164 kb) [file 12864_2018_4583_MOESM4_ESM.pdf]

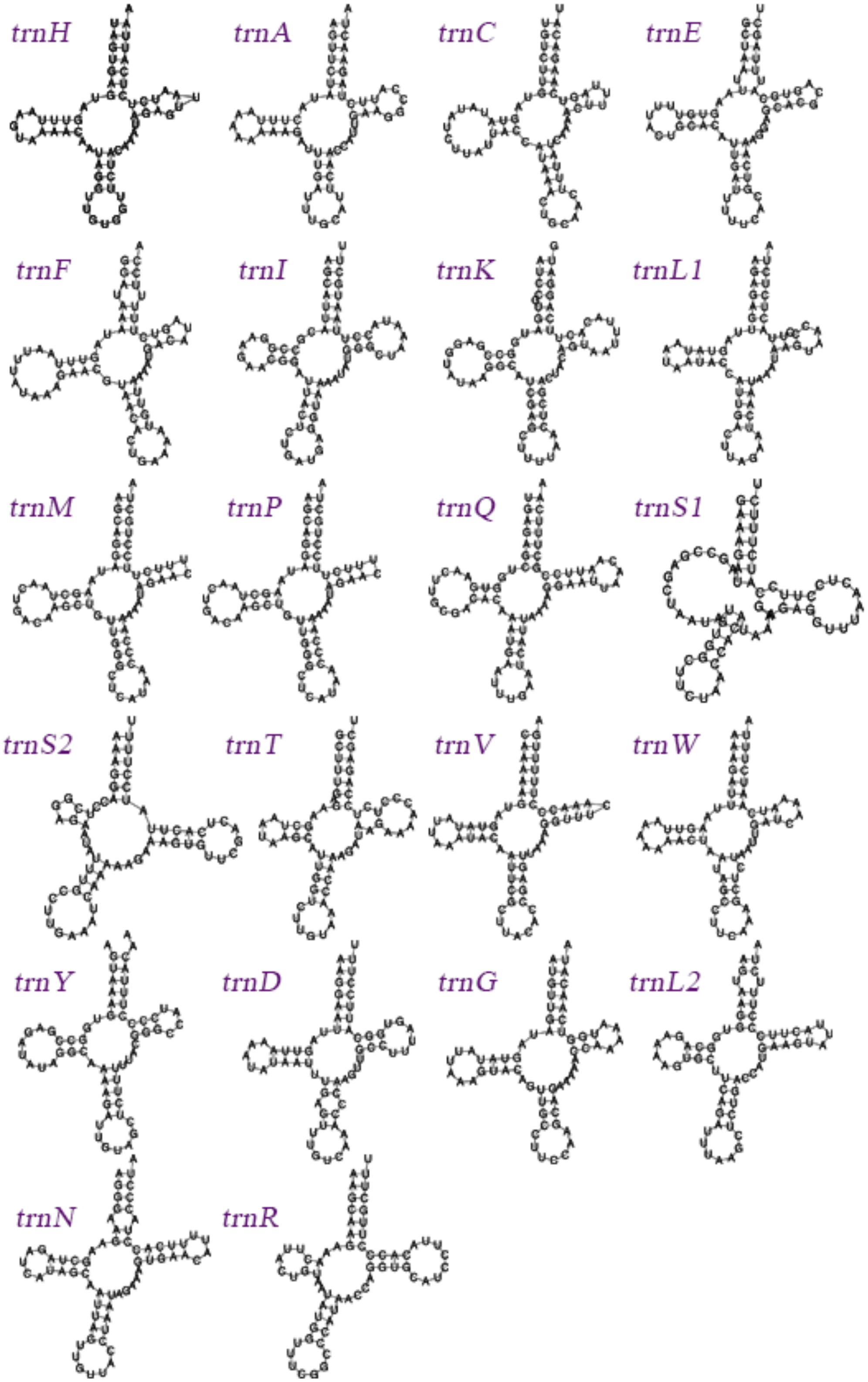

Additional File 4. Predicted tRNA structures for all 22 tRNA of H-type mitochondrial DNA in *A. cygnea*. Left-to-right and top-to-bottom order follows their placement within the annotated complete mitochondrial genome beginning at base 2,668.
