# Supplementary material for: The complete mitochondrial genome of the hermaphroditic freshwater mussel Anodonta cygnea (Bivalvia: Unionidae): in silico analyses of sex-specific ORFs across order Unionoida
Source: BMC Genomics. 2018 Mar 27;19:221. doi: 10.1186/s12864-018-4583-3 (PMC5870820; doi:10.1186/s12864-018-4583-3)
Supplement: Supplementary file 5 — Figure S2. Comparative analysis of an F- and H-ORF of closely related species. (A) Kyte-Doolittle hydrophobicity plots of the Anodonta anatina F-ORF (GenBank Accession YP008802631) and Anodonta cygnea H-ORF (this study). (B) Amino acid alignment of the same A. anatina F-ORF and A. cygnea H-ORF, where black represents the same amino acid, grey is similar and white is different. (PDF 116 kb) [file 12864_2018_4583_MOESM5_ESM.pdf]

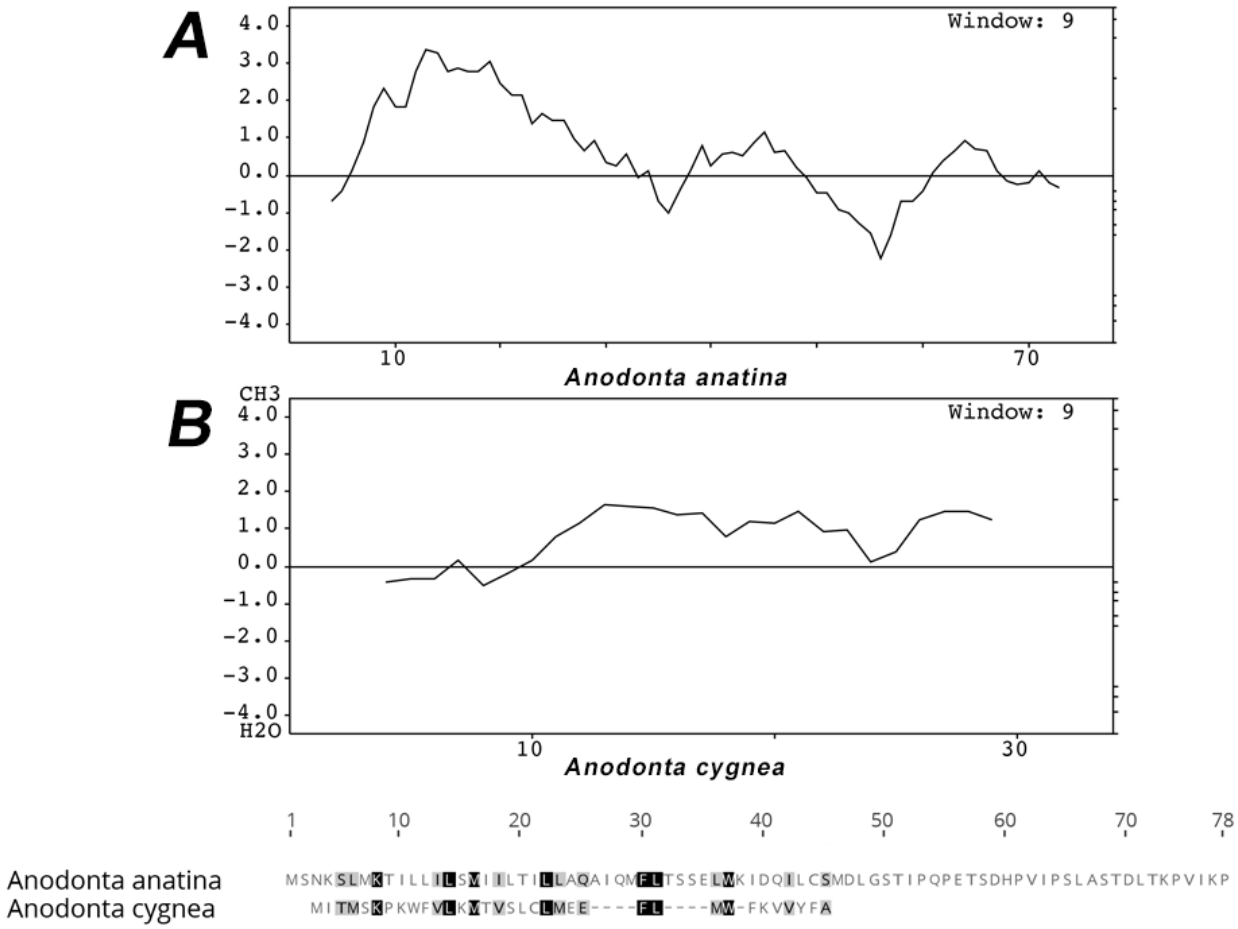

Additional File 5. Comparative analysis of an F- and H-ORF of closely related species. (A) Kyte-Doolittle hydrophobicity plots of the *Anodonta anatina* F-ORF (GenBank Accession YP008802631) and *Anodonta cygnea* H-ORF (this study). (B) Amino acid alignment of the same *A. anatina* F-ORF and *A. cygnea* H-ORF, where black represents the same amino acid, grey is similar and white is different.
