# Supplementary material for: The complete mitochondrial genome of the hermaphroditic freshwater mussel Anodonta cygnea (Bivalvia: Unionidae): in silico analyses of sex-specific ORFs across order Unionoida
Source: BMC Genomics. 2018 Mar 27;19:221. doi: 10.1186/s12864-018-4583-3 (PMC5870820; doi:10.1186/s12864-018-4583-3)
Supplement: Supplementary file 6 — Table S4. Sequence divergence of nad5-trnQ between H-type and closely related F-type mitochondrial DNA. (PDF 43 kb) [file 12864_2018_4583_MOESM6_ESM.pdf]

Additional File 6. Sequence divergence of the unassigned region *nad5-trnQ* between H-type mitochondrial genomes and a closely related F-type mitochondrial genome. Alignments performed in Geneious 11.0.3 using default parameters with an IUB cost matrix.

H-type taxa	F-type taxa	Percent sequence Divergence
Anodonta cygnea	*Anodonta anatina*	57.5
Utterbackia imbecillis	*Utterbackia peninsularis*	60.0
Margaritifera falcata	*Cumberlandia monodonta*	65.7
Lasmigona compressa	*Pyganodon grandis*	56.6
Toxolasma parvus	*Venustaconcha ellipsiformis*	48.2
