# Supplementary material for: The complete mitochondrial genome of the hermaphroditic freshwater mussel Anodonta cygnea (Bivalvia: Unionidae): in silico analyses of sex-specific ORFs across order Unionoida
Source: BMC Genomics. 2018 Mar 27;19:221. doi: 10.1186/s12864-018-4583-3 (PMC5870820; doi:10.1186/s12864-018-4583-3)
Supplement: Supplementary file 7 — Figure S3. NAD5 amino acid alignment of Anodonta cygnea and Anodonta anatina. (PDF 84 kb) [file 12864_2018_4583_MOESM7_ESM.pdf]

A

```
Anodonta_cygnea       MKNKKKVSVPLLCSGFLFFTSLFLWVFGVYMVGSVGCSYMVEWQFFSLCSTDWSLPITID      60
Anodonta_anatina      MKNKKKMSVPLLCSVSFFFIALLLWVFGVYMIGSTGRSYMVEWQFFSLCSSDWALPVIID      60
                      ******:*******   :** :*:********:**.* *************:**:**: **

Anodonta_cygnea       YISVIFSCFVCLISGSVSLFSVSYMEEEVFMRRFMMLIMAFVGSMNMLIYIPSLITILLG      120
Anodonta_anatina      YISIIFSCFVCLISGSVSLFSVSYMEGEVFMRRFMLLIVAFVGSMNLLIFIPNLITILLG      120
                      ***:********************** ********:**:*******:**:**.*******

Anodonta_cygnea       WDGLGIVSFALVIYYQNKKSLAAGMLTVLANRIGDALLILSICFLVNWGEWRIGYGMSGS      180
Anodonta_anatina      WDGLGIVSFALVIYYQNKKSLAAGMLTVLANRIGDVLLILSICFLVNWGEWRIGYGMTGS      180
                      ***********************************.********************:**

Anodonta_cygnea       FSLVICFLVVVGAMTKSAQIPFSAWLPAAMAAPTPVSALVHSSTLVTAGVYLVIRFYSTL      240
Anodonta_anatina      FSMVICFLVVVGAMTKSAQIPFSAWLPAAMAAPTPVSALVHSSTLVTAGVYLIIRFYSTL      240
                      **:*************************************************:*******

Anodonta_cygnea       IEAQEVLWFLSKVGALTLLMAGLSACFEVDLKKIIALSTLSQLGLMMFTVGIGFPLIAVF      300
Anodonta_anatina      IETQEVLWFLSKIGALTLLMAGLSACFEVDLKKIIALSTLSQLGLMMFTVGIGFPLIAVF      300
                      **:*********:***********************************************

Anodonta_cygnea       HLLTHALFKALLFLCAGSIIHSTMDTQDGRILGSLNYLLPFSSGCLVLSSVALCGMPFLS      360
Anodonta_anatina      HLLTHALFKALLFLCAGSIIHSTMDTQDGRILGSLNYLLPYSSSCLVLSSVVLCGMPFLS      360
                      ****************************************:**.*******.********

Anodonta_cygnea       GFYSKDLILEGAFSGFSGSLEILVMLVGAGLSLVYSLRIMLIGVFGQNYSSSLVSYGVEG      420
Anodonta_anatina      GFYSKDLILEGAFSGFSGSLEVLVMSMGAGLSLIYSLRILLVGVFGQNYSSSLVNYSAES      420
                      *********************:***  :******:*****:*:***********.*..*.

Anodonta_cygnea       VMWCLLFV--S-------   CQLVQLWEGDY-FKEYGLM-----------------------      447
Anodonta_anatina      GYVVSSIMILSVGAISGGWLLQSVWVGVNGFSLIGVLGKVVISVVTFVGLVYGFINYFMA      480
                            ::  *          * .:* *   *.  *::

Anodonta_cygnea       ------------------------------------------------------------      447
Anodonta_anatina      GILGEKSFSGLGRFLSSMWFMNLTSGSFLAGIGLKLGKMMHLHLDLGWMEVLGGQGVFSV      540

Anodonta_cygnea       --------------------------------------      447
Anodonta_anatina      LGDGVKMSYLMQSGSLLMHTRTYMVLMMTVLIMGVYLL      578
```

B

```
Anodonta_anatina      MKNKKKMSVPLLCSVSFFFIALLLWVFGVYMIGSTGRSYMVEWQFFSLCSSDWALPVIID      60
ptNAD5                ------------------------------------------------------------      0

Anodonta_anatina      YISIIFSCFVCLISGSVSLFSVSYMEGEVFMRRFMLLIVAFVGSMNLLIFIPNLITILLG      120
ptNAD5                ------------------------------------------------------------      0

Anodonta_anatina      WDGLGIVSFALVIYYQNKKSLAAGMLTVLANRIGDVLLILSICFLVNWGEWRIGYGMTGS      180
ptNAD5                ------------------------------------------------------------      0

Anodonta_anatina      FSMVICFLVVVGAMTKSAQIPFSAWLPAAMAAPTPVSALVHSSTLVTAGVYLIIRFYSTL      240
ptNAD5                ------------------------------------------------------------      0

Anodonta_anatina      IETQEVLWFLSKIGALTLLMAGLSACFEVDLKKIIALSTLSQLGLMMFTVGIGFPLIAVF      300
ptNAD5                ------------------------------------------------------------      0

Anodonta_anatina      HLLTHALFKALLFLCAGSIIHSTMDTQDGRILGSLNYLLPYSSSCLVLSSVVLCGMPFLS      360
ptNAD5                ------------------------------------------------------------      0

Anodonta_anatina      GFYSKDLILEGAFSGFSGSLEVLVMSMGAGLSLIYSLRILLVGVFGQNYSSSLVNYSAES      420
ptNAD5                ------------------------------------------------------------      0

Anodonta_anatina      GYVVSSIMILSVGAISGGWLLQSVWVGVNGFSLIGVLGKVVISVVTFVGLVYGFINYFMA      480
ptNAD5                --------------------------------MVGVLGKGIISVVTFVGLSYGVFSYFML      28
                                                       ::***** :********* **.:.***

Anodonta_anatina      GILGEKSFSG-LGRFLSSMWFMNLTSGSFLAGIGLKLGKMMHLHLDLGWMEVLGGQGVFS      539
ptNAD5                EAFKKKVFKGNLGWFLSSMWFMNLTSGSFLAGWG--------------------------      62
                        : :* *.* ** ***************** *

Anodonta_anatina      VLGDGVKMSYLMQSGSLLMHTRTYMVLMMTVLIMGVYLL      578
ptNAD5                ---------------------------------------      62
```

Additional File 7. (A) Amino acid alignment of *Anodonta cygnea* NAD5 (MF781083) and *Anodonta anatina* NAD5 (KF030964). (B) Amino acid alignment of *A. anatina* (KF030964) NAD5 with the putative translocated NAD5 from *A. cygnea* (MF781083). Alignments conducted using Clustal Omega webserver with default parameters. An asterisk (*) indicates a fully conserved residue, a colon (:) indicates residues with strongly similar properties, and a period (.) indicates weakly similar properties.
