# Supplementary material for: The complete mitochondrial genome of the hermaphroditic freshwater mussel Anodonta cygnea (Bivalvia: Unionidae): in silico analyses of sex-specific ORFs across order Unionoida
Source: BMC Genomics. 2018 Mar 27;19:221. doi: 10.1186/s12864-018-4583-3 (PMC5870820; doi:10.1186/s12864-018-4583-3)
Supplement: Supplementary file 8 — Figure S4. Nucleotide alignment of three translocated portions of nad5 from three Anodonta cygnea individuals. A.cygnea 3 is the subject of this paper (Genbank accession MF781083). (PDF 70 kb) [file 12864_2018_4583_MOESM8_ESM.pdf]

```
Anodonta_cygnea_1      ACCTCCCAGACGTCAAATTTATAAACCACATTCTAGATAAAAACCAACCTAAATTACCTT      60
Anodonta_cygnea_2      ACCTCCCAGACGTCAAATTTATAAACCACATTCTAGATAAAAACCAACCTAAATTACCTT      60
Anodonta_cygnea_3      ACCTCCCAGACGTCAAATTTATAAACCACATTCTAGATAAAAACCAACCTAAATTACCTT      60
                       ************************************************************

Anodonta_cygnea_1      TAAAAACCTTTTTTTTGAAGGCTTCTAATATGAAATAACTAAACACCCCATAGGACAAAC      120
Anodonta_cygnea_2      TAAAAACCTTTTTTTTGAAGGCTTCTAATATGAAATAACTAAACACCCCATAGGACAAAC      120
Anodonta_cygnea_3      TAAAAACCTTTTTTTTGAAGGCTTCTAATATGAAATAACTAAACACCCCATAGGACAAAC      120
                       ************************************************************

Anodonta_cygnea_1      CTACAAACGTTACAACCCTAATAATTCCCTTTCCCAAAACACCTACCAACCTAAACCCAT      180
Anodonta_cygnea_2      CTACAAACGTTACAACCCTAATAATTCCCTTTCCCAAAACACCTACCAACCTAAACCCAT      180
Anodonta_cygnea_3      CTACAAACGTTACAACCCTAATAATTCCCTTTCCCAAAACACCTACCAACCTAAACCCAT      180
                       ************************************************************

Anodonta_cygnea_1      TTACATCAACCCATAC-     196
Anodonta_cygnea_2      TTACATCAACCCATAC-     196
Anodonta_cygnea_3      TTACATCAACCCATACT     197
                       ****************
```

Additional File 8. Nucleotide alignment of three translocated portions of *nad5* from three *Anodonta cygnea* individuals. *A*. *cygnea* 3 is the subject of this paper (Genbank accession MF781083).
